# Impact of Neem Seed Extract on Mortality, Esterase and Glutathione-S-Transferase Activities in Thai Polyvoltine Hybrid Silkworm, *Bombyx mori* L.

**DOI:** 10.3390/insects15080591

**Published:** 2024-08-03

**Authors:** Ajin Rattanapan, Patcharawan Sujayanont

**Affiliations:** 1 Department of Biology, Faculty of Science, Mahasarakham University, Kantharawichai District, Mahasarakham 44150, Thailand; 2 Center of Excellence for Mulberry and Silk, Mahasarakham University, Kantharawichai District, Mahasarakham 44150, Thailand; 3 Department of Preclinic, Faculty of Medicine, Mahasarakham University, Muang District, Mahasarakham 44000, Thailand; 4 Tropical Health Innovation Research Unit, Mahasarakham University, Muang District, Mahasarakham 44000, Thailand

**Keywords:** silkworm, *Bombyx mori*, neem seed extract, biopesticide toxicity, esterase, glutathione-S-transferase

## Abstract

**Simple Summary:**

Thailand’s polyvoltine hybrid silkworm, Dok Bua, is 30% more productive than indigenous breeds and is robust and well-suited to the local environment, making it a preferred choice in Thai sericulture. However, synthetic insecticides used on mulberry pests pose risks to silkworms. Neem extract tends to be a safer alternative. The potential toxicity of Thai neem seed extract on this silkworm and their enzymatic detoxification capability could substantially affect their growth and productivity. To explore this, we carried out toxicological and biochemical assessments. The results indicated that the extract had a significant toxicity across all larval stages. Younger larvae were notably more susceptible. We proceeded to examine the activity of the key enzymes implicated in xenobiotic metabolism. The finding that the detoxification enzyme had a low level of activity in the early instar larvae aligned with the larvae mortality. Neem extract suppressed esterase activity but induced glutathione S-transferases (GST) activity, remarkably, in the whole body. Despite its toxicity, GST metabolism could mitigate the effects, indicating the detoxification capabilities of silkworms. These novel findings suggest that neem extract is toxic to all larvae, with GST playing a critical role in detoxification, highlighting the need for careful consideration in sustainable sericulture safety practices.

**Abstract:**

Neem, a biopesticide, offers a safer alternative to the synthetic insecticides commonly used in mulberry cultivation, which can harm silkworms. This study aimed to investigate the effects of Thai neem seed extract on all instar larvae of the Thai polyvoltine hybrid silkworm, *Bombyx mori* L., Dok Bua strains, focusing on the mortality rate and the activities of esterase (EST) and glutathione S-transferases (GST) enzymes. Acute toxicity was assessed using the leaf-dipping method. Results showed that the mortality rate tended to be higher in younger instars than in older ones. The first instar larvae exhibited the highest mortality rate at 94%, whereas the LC_50_ was highest in the third instar at 5.23 mg L^−1^ at 72 h. This trend aligns with the activities of EST and GST, which were evaluated in the whole bodies of the first instar larvae and the midgut tissue of fifth instar larvae. As the extract concentration increased, EST activity decreased while GST activity increased in both the first and fifth instar larvae. These findings highlight that neem extract is toxic to all instar larvae, with GST playing a crucial role in detoxification, particularly in the whole body of the Thai polyvoltine hybrid silkworm.

## 1. Introduction

Silkworms (*Bombyx mori* L.) produce luxurious silk with both cultural and economic value [1,2,3]. In Thailand, the Ubon Ratchathani 60-35 (Dok Bua) hybrid silkworm strains have been developed to enhance productivity, showing a 30% increase in yields compared to native strains. This strain is noted for its shorter larval lifespan, reduced production costs, and lower disease risk. It is robust, easy to cultivate, and well suited to the Thai environment, producing medium-sized cocoons with high-quality yellow filament [4]. Silkworms are monophagous insects, feeding exclusively on mulberry leaves, and their development is closely linked to the quality of the leaves [5,6]. Insecticides used to control mulberry pests can negatively impact silkworms [7,8,9]. Silkworms are vulnerable to pesticides, which can cause poisoning and other detrimental effects [10,11]. Finding new insecticides that are both effective against mulberry pests and safe for silkworms is of paramount importance. The development of botanical insecticides is rapidly expanding, fueled by the need to find alternatives to synthetic chemical insecticides, which have significant harmful effects. Therefore, developing safe and effective pest control methods like plant-based insecticides is critical for sustainable sericulture [12,13].

Neem is a promising bioinsecticide that interrupts insect growth and offers an eco-friendly alternative to synthetic insecticides. Extensive research into neem is recommended to fully harness its potential. Neem extracts and products can serve as a safe and effective alternative to synthetic biocides in pest and disease management, especially for low-income farmers [14]. Due to neem being a native plant in tropical regions, farmers can produce it by themselves and, for Thai farmers as well, it can be carried out easily and inexpensively. In many rural areas of Thailand and other countries, neem seed extract is commonly prepared using water as a solvent due to its availability and safety. The dosage of aqueous extract used as a biopesticide or for other agricultural purposes varies depending on the target pest, crop, and local practices. The extract is used either directly or diluted with water to achieve the desired concentration for practical applications [15,16,17,18,19]. However, the concentration is not specifically documented, posing a risk of toxicity to non-target insects, particularly economically important insects such as silkworms. Several neem parts are insecticide-effective; the seed kernels of neem contain several limonoids, which exert growth regulation on various insect pests [20]. It is also an effective insect management option in mulberry production [21]. While neem extract is beneficial for mulberry cultivation, it has been associated with the inhibition of growth and cocooning or decreased silk quality as well [22]. Therefore, further information on the potential adverse effects of neem extract on economically significant beneficial insects, such as silkworms, is essential.

Insects have developed a range of detoxification mechanisms to resist the toxic effects of xenobiotics including plant toxins. Identifying the detoxification enzymes responsible for metabolizing insecticides is crucial for planning effective pest control strategies and minimizing the harmful effects on non-target organisms. These mechanisms involve the upregulation of several enzyme families in phase I and phase II reactions that play a crucial role in metabolizing various toxins and assisting in removing xenobiotics [23]. In phase I reactions, esterase (EST) acts against a broad range of chemical classes which are catalysts that facilitate the hydrolysis of ester bonds [24]. Glutathione-S-transferases (GST) are multifunctional enzymes in phase II reactions that facilitate the solubility and removal of toxins [25]. In recent years, scholarly attention has become increasingly focused on understanding how silkworms react to insecticides and plant-derived compounds with potential pesticidal properties. Some studies have examined the effects of bioagents on silkworm detoxification expression. For instance, quercetin might inhibit silkworm detoxifying enzymes, resulting in higher mortality rates [26]. A fresh understanding of silkworm cytosolic sulfotransferases ST3 aids in polyphenol detox in silkworm midguts [27]. The Meliaceae limonoids such as azadirachtin interact with gut enzymes, potentially impacting enzyme expression, leading to significant disruptions in insect growth and development [28]. This interaction is particularly useful for managing both insect pests and the benefits that depend on enzyme-based metabolism. Despite observing the efficacy of these detoxifying enzymes in silkworms against insecticides from the aforementioned report, clear information on the role of detoxifying enzymes against neem extracts in Thai silkworm strains remains lacking, especially in the hybrid strains. It is imperative to conduct this study because different silkworm strains have varying abilities to employ detoxifying enzymes against various insecticides. The expression and activity levels of detoxification enzymes can vary significantly among insect populations and strains, contributing to differences in insecticide susceptibility [29,30]. Strain-specific differences in the detoxification capabilities of each silkworm strain against various insecticides have been reported [31,32]. The biochemical responses of the silkworm aid in evaluating the potential risks and advantages of using neem in sericulture thus contributing to the development of safer pest management strategies.

Therefore, to address this challenge in sericulture, this study aims to bridge this gap by evaluating the toxicity of Thai neem seed extract and its effects on some important detoxification enzymes in Thai hybrid silkworms, specifically the Dok Bua strains. The findings of this study will provide insights into effectively mitigating the phytochemical toxicity of neem extract to silkworms and contribute to understanding the biochemical responses of silkworms to neem-based insecticides. Consequently, this will help reduce yield damage and promote the development of sustainable sericulture practices, thereby supporting the sustainable development of the sericulture industry.

## 2. Materials and Methods

### 2.1. Silkworm Larvae Rearing

The eggs of the Thai hybrid silkworm strains (Ubon Ratchathani 60-35; Dok Bua) were obtained from the Queen Sirikit Department of Sericulture. Larvae were reared under conditions of 25–27 °C, 70–80% relative humidity, and day/night period of 12:12 h in the laboratory of the Biology Department, Faculty of Science, Mahasarakham University, Thailand. Silkworm larvae were fed with fresh, clean, and pesticide-free mulberry leaves thrice daily, following a modified method from Nonsrirach et al. [33]. With the same sizes, two-day-old larvae from the first to fifth instar stages were collected for toxicity evaluation. For the next experiment, larvae at the first and fifth instar larvae stages were gathered to explore the detoxification enzyme mechanisms, namely the activities of esterase and glutathione-S-transferase.

### 2.2. Neem Seed Extract Preparation

Mature neem seeds were collected from a disease-free tree located at 17°15′37.88″ N, 102°53′17.50″ E in the Udon Thani province, Thailand. The plant specimen, *Azadirachta indica* A.Juss., was archived at the Department of Biology, Faculty of Science, Mahasarakham University, and catalogued as voucher specimen number MSUT-8477. Raw seeds were cleaned, and the seed coat was removed from the kernels. After drying the seeds in a Hot Air Oven (Memmert-600) at 60 °C for 48 h, they were ground into a fine powder using an electric grinder, Mxbaoheng Instrument Company, China. Neem seed powder was sequentially extracted by macerating it with distilled water at a 1:4 ratio for a week at room temperature (36–37 °C), following a modified method from Tabassam et al. [34]. The aqueous extract was filtered through Whatman filter paper No.1 and evaporated using a rotary evaporator. The crude extract was then freeze-dried and stored at 4 °C until the experiment commenced.

### 2.3. Toxicity Bioassay

The toxicity assessment was conducted using a completely randomized design with five replicates, each containing 60 silkworm larvae under identical laboratory conditions described for insect rearing. This study focused on the toxicity of neem seed crude extract on the silkworm larvae at the first to fifth instar. Aqueous extract was prepared for the bioassay with deionized water (DI H_2_O) into four concentrations—5, 15, 25, and 35 mg L^−1^. A leaf-dipping method, modified from Shao et al. and Chen et al. [35,36], was employed in the assay. Fresh and healthy mulberry leaves were cleaned with distilled water, air dried, and cut into discs of 3 cm in diameter. Each disc was immersed in the extract for 1 min and then air-dried on filter paper at room temperature. One treated mulberry leaf disc was placed in a glass Petri dish, with 60 larvae in each replicate for the first and third instar. In the third to fifth instars, the larvae were placed individually in plastic boxes, one larva per box fed on the leaf disc. Subsequently, each replication of silkworms was reared together in a silkworm rearing basket. The treated discs were provided to the silkworms during the morning feeding on the second day of each instar, followed by feeding with healthy and non-toxic foliage. Control group silkworms were fed with deionized water (DI H_2_O)-treated mulberry leaves. Mortality was recorded at 24, 48, and 72 h after exposure to determine the toxicity values. The larvae were considered dead if they did not move when probed with a brush. The data were analyzed using a standard probit analysis [37].

### 2.4. Detoxification Enzyme Activity Estimation

#### 2.4.1. Enzyme Extraction

The activities of two critical enzymes involved in phase I and II metabolism, namely EST and GST, were assessed. Enzyme assays were conducted in vivo, targeting the whole body and the midgut of two larval stages with varying toxin tolerance, specifically the first and fifth instar silkworms, respectively. The extraction followed the specified biological testing protocols for both treated and control groups, with each concentration tested in triplicate. After 24 h exposure, surviving larvae were utilized for enzyme extraction to evaluate enzyme activities. Enzymes from each larval group were prepared in ice-cold DI H_2_O. The extraction procedure for the whole bodies of the first instar larvae adhered to the methodologies described by Phairiron and Yooboon et al. [38,39]. The isolation of midgut tissues from the fifth instar larvae was carried out according to a modified method by Wang et al. [40]. Midguts were dissected individually, with three midguts per sample (0.16 g tissue per insect). Depending on the sample type, either 0.5 g of whole-body surviving larvae or midgut tissues were homogenized in 1 mL of 0.1 M potassium phosphate buffer containing 1 mM EDTA at pH 7.8, followed by 10,000× *g* for 5 min at 4 °C. The resulting supernatant was transferred to a clean 1.5 mL microtube and placed on ice for immediate enzyme activity determination.

#### 2.4.2. Estimation of Enzyme Activities and Protein Content

(1) Esterase Enzyme (EST)

EST activity was measured using a modified assay of p-nitrophenyl acetate (pNPA), as described by Phairiron and Simplício et al. [38,41]. The substrate was dissolved in phosphate buffer, pH 7, to obtain 1 mM pNPA solution. The reaction mixture comprised 900 µL of phosphate buffer, pH 7, and 50 µL of the enzyme extract. The mixture cuvette was incubated at 25 °C for 5 min to achieve the required temperature. Subsequently, 50 µL pNPA solution was added to the cuvette and mixed thoroughly. The measurement of the absorbance was carried out using a spectrophotometer, Thermo Fisher Scientific, China, at 405 nm. The change in absorbance per minute (ΔA405/min) was determined from the linear portion of the absorbance–time curve. EST activity was expressed as nmol of p-nitrophenol released per minute per mg protein, and the extinction coefficient of p-nitrophenol (ε = 18,000 mM^−1^ cm^−1^ at 405 nm) was used for the calculation. Control samples excluded the enzyme sample.

(2) Glutathione S-Transferase Enzyme (GST)

GST activity was characterized using 1-chloro-2,4-dinitrobenzene (CDNB) and 1 mM GSH as standard substrates. The modified assays by Habig et al. and Yamamoto and Yamada [27,42] were used. CDNB was dissolved in ethanol to make a 20 mM stock solution. Reduced glutathione (GSH) was prepared in DI H_2_O for a 20 mM stock solution. The spectrophotometer and all the reagents were prewarmed to 25 °C. The reaction mixture was prepared from 2.7 mL phosphate buffer (0.1 M, pH 8) mixed with 0.1 mL GSH solution and 0.1 mL enzyme source. The mixed solution was incubated at 25 °C for 5 min in the spectrophotometer. Then, 0.1 mL CDNB substrate was added and mixed quickly, after which the absorbance was immediately measured at 340nm for 5 min at 25 °C. The increase in absorbance was due to the formation of the conjugate between GSH and CDNB. The change in absorbance per minute (ΔA340/min) was calculated from the linear portion of the curve. The GST activity was determined using the extinction coefficient of the CDNB–GSH conjugate (9.6 mM^−1^cm^−1^) and expressed as nmol per min per mg protein. The control excluded enzyme extract.

(3) Total Protein Content

The Bradford method was performed to determine the total protein levels [43], with bovine serum albumin as a standard. The measurements were observed at 595 nm absorbance.

### 2.5. Data Analysis

All variables were succinctly summarized through the calculation of means and standard deviations. Discrepancies among the mortality rates of larvae exposed to different concentrations of the extract at 24, 48, and 72 h were examined utilizing a two-way analysis of variance (ANOVA), followed by Tukey’s post hoc tests for pairwise comparisons. A Probit regression analysis was conducted utilizing BioRssay [44] to determine the lethal concentrations (LC_10_, LC_50_, and LC_90_) and their corresponding 95% confidence intervals (CI). Differences in dose–mortality responses between the various larval stages were juxtaposed in pairs using Bonferroni correction to address multiple comparisons. The assessment of variations in the EST and GST activities and protein content was carried out using a one-way ANOVA test, followed by Tukey’s HSD post hoc analysis. A significance level of *p* < 0.05 was established for statistical significance. The statistical analyses were conducted utilizing the R version 4.1.0 software package.

## 3. Results

### 3.1. Effects of Thai Neem Seed Crude Extract on Thai Hybrid Silkworm Larvae

#### 3.1.1. Effect on Silkworm Larvae Mortality

**Figure 1:**
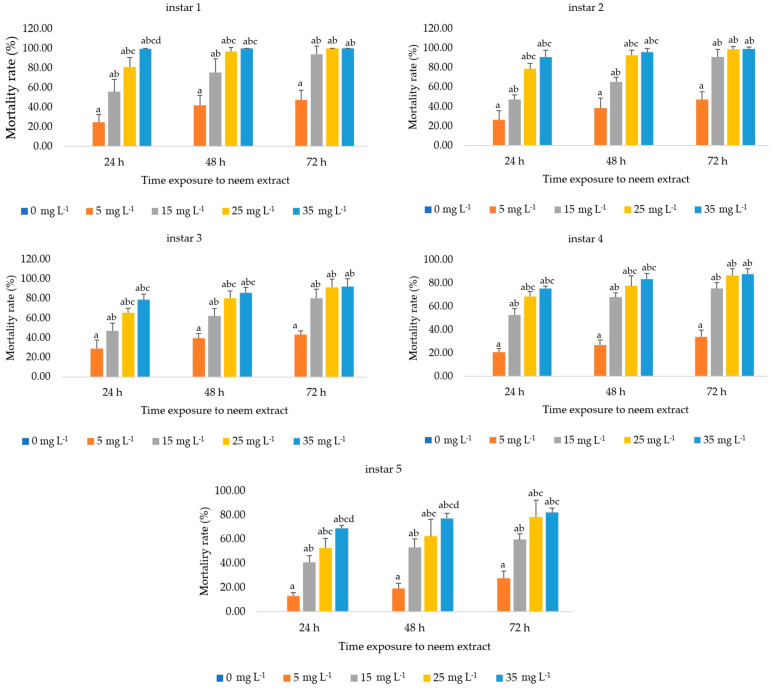
Mortality rates of first to fifth instar *Bombyx mori* larvae after exposure to various concentrations of neem extracts (0, 5, 15, 25 and 35 mg L^−1^) at 24, 48 and 72 h. The bar graph represents mean ± SD. Differences between groups were analyzed using two-way ANOVA, followed by Tukey’s HSD post hoc analysis for multiple comparisons. Significant differences between groups are indicated by *p*-values < 0.05. Letters a, b, c and d represent the difference between the given neem extract concentration and the concentration of 0, 5, 15 and 25 mg L^−1^, respectively.

The acute toxicity of the aqueous extract was evaluated. The mortality rate of each instar larvae had significant differences across the different extract concentrations and the durations of exposure. The mortality rate progressively decreased in later instar silkworms. The results showed that the extract exhibited the highest significant mortality (*p* < 0.05) in the first instar larvae after 24 h of exposure to 15 mg L^−1^, with a mortality rate of over 50% (56 ± 12.51%). The increase rose to 94 ± 8.30% at 72 h after treatment under the same conditions. Additionally, larval mortality reached 100% at 72 h post-treatment with 25 mg L^−1^. The mortality rates of the second to fifth instar larvae followed the same trend. They increased significantly with both time of exposure and concentration of neem extract. By 72 h, the mortality rates in higher concentrations converged, showing no significant differences among them, but all were higher than the control. The second, third, and fifth instar larvae exhibited mortality rates exceeding 50% but not reaching 100% when exposed to concentrations of 15 mg L^−1^ or higher starting from 48 h onwards. The fourth instar larvae exhibited mortality rates exceeding 50% but not reaching 100% when exposed to concentrations of 15 mg L^−1^ or higher starting from 24 h onwards, whereas the fifth instar larvae exhibited the lowest mortality rate, with 13.33 ± 2.64% after 24 h of exposure to 5 mg L^−1^ extract. However, no deaths occurred in the control group, as shown in Figure 1. The experimental results showed that all the instar larvae of silkworms exhibited sensitivity to neem extract, with particular susceptibility observed in early instar larvae.

#### 3.1.2. Toxicity of Neem Seed Extract on Silkworm Larvae

**Figure 2:**
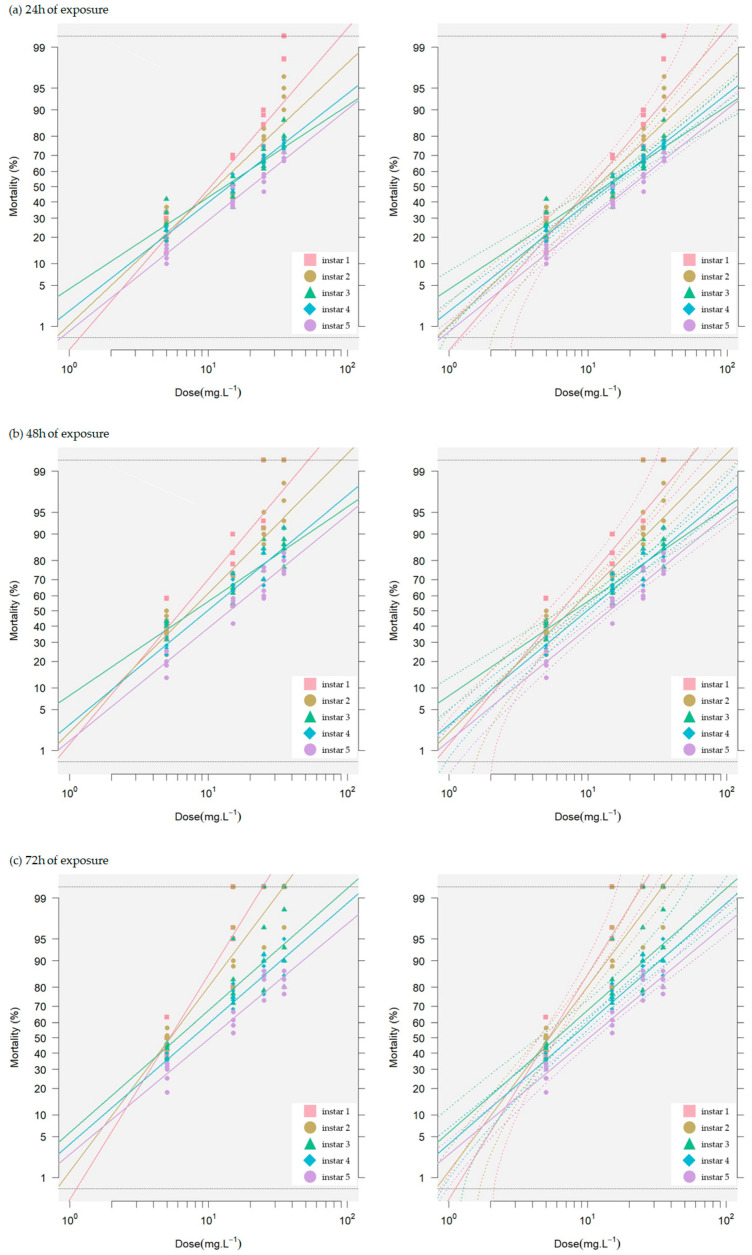
Linear relationships between probit-transformed mortality rates and the log-dose of neem extracts for different instars of *Bombyx mori* larvae. The left panels show the linear regressions, while the right panels include 95% confidence intervals. (**a**) 24 h of exposure. (**b**) 48 h of exposure. (**c**) 72 h of exposure.

The extract demonstrated toxicity as indicated by LC_10_, LC_50_, and LC_90_ values. For LC_10_, it was significantly highly toxic (*p* < 0.05) to the third instar larvae at a concentration of 1.21 mg L^−1^ after 48 h. In comparison, the LC_50_ and LC_90_ values showed a high effect on the first instar larvae at 72 h with concentrations of 5.23 and 12 mg L^−1^ extract, respectively. All experiments showed low toxic effects on the fifth instar larvae at 24 h, with LC_10_, LC_50_, and LC_90_ values of 4.1, 20, and 99 mg L^−1^, respectively (Table 1 and Figure 2). The toxicity values of all instar larvae in terms of LC_10_, LC_50_, and LC_90_ at various exposure times are shown in Table 1. The differential response to exposure times for each instar is illustrated in Figure 2. The LC_50_ value of the extract was highest when each instar larva was exposed to the maximum concentration of 35 mg L^−1^ for the longest duration of 72 h. These values for the first to fifth instar larvae were 5.23, 5.29, 5.96, 7.61, and 10.00 mg L^−1^, respectively. The results indicate that neem seed crude extract is highly toxic to early instar silkworms. It was 1.91 times more toxic to the first instar larvae than to the fifth instar larvae. However, the findings suggest that the extract has a pronounced toxic effect on all larval stages of the Dok Bua silkworm strain.

### 3.2. Estimation of Detoxification Enzyme Activity

The study revealed that Thai neem seed crude extract significantly affects the detoxification enzymes in silkworm larvae. Increasing extract concentrations led to decreased esterase (EST) activity in both the first instar whole body and fifth instar midgut tissue, with greater suppression in the whole body. Conversely, glutathione-S-transferase (GST) activity increased with higher extract concentrations, showing a greater induction in the whole body compared to midgut tissue. These findings demonstrate the extract’s differential impact on key detoxification enzymes in silkworms. The results are presented below.

#### 3.2.1. Estimation of Esterase Enzyme (EST)

**Figure 3:**
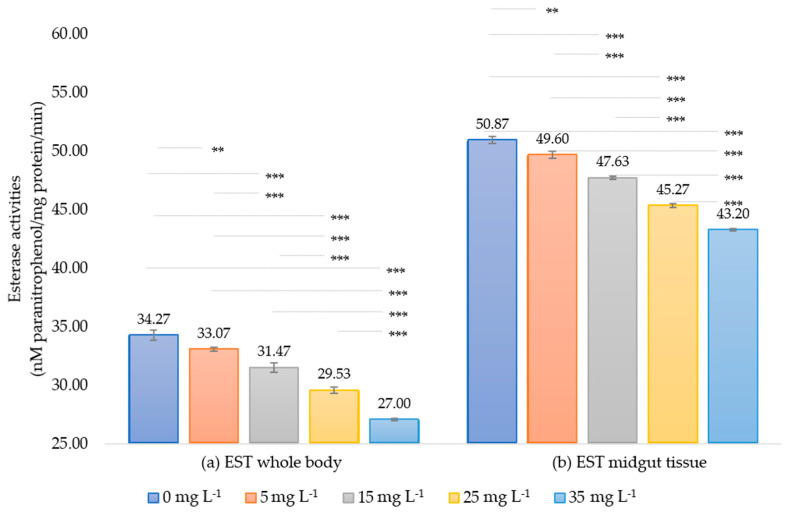
Esterase enzyme activity (nmol para-nitrophenol/mg protein/min) in the whole larvae body of first instar (**a**) and midgut tissue of fifth instar (**b**) of *Bombyx mori* after 24 h of exposure to various concentrations of neem extracts. The bar graph represents means ± SD. Differences between groups were analyzed using one-way ANOVA, followed by Tukey’s HSD post hoc analysis for multiple comparisons. Significant differences between groups are represented as ** and *** for *p*-values < 0.01 and < 0.001, respectively.

The enzymatic activities in all extract treatments were reduced compared to the control. The whole-body EST activities of the first instar larvae were recorded as 34.27 ± 0.42, 33.07 ± 0.15, 31.47 ± 0.25, 29.53 ± 0.25, and 27.00 ± 0.10 nmol para-nitrophenol/mg protein/min when exposed to 0, 5, 15, 25, and 35 mg L^−1^ of extract, respectively. These values indicate relative activity reductions of 0.96, 0.92, 0.86, and 0.79-fold compared to the control. In the midgut tissue of fifth instar larvae, the activities were 50.87 ± 0.31, 49.60 ± 0.30, 47.63 ± 0.12, 45.27 ± 0.15, and 43.20 ± 0.10 nmol para-nitrophenol/mg protein/min at the same extract concentrations, respectively. These values correspond to the relative activity decreases of 0.98, 0.94, 0.89, and 0.85-fold compared to the control (Figure 3). When exposed to the neem seed extract, the data reveal significant reductions in EST activities (*p* < 0.05) in both enzyme sources of silkworm larvae. Moreover, there were significant differences in enzyme activity between the highest (35 mg L^−1^) and the lowest (5 mg L^−1^) extract concentrations, with EST activity being more suppressed in the whole body than in the midgut tissue.

#### 3.2.2. Estimation of Glutathione-S-Transferase Enzyme (GST)

**Figure 4:**
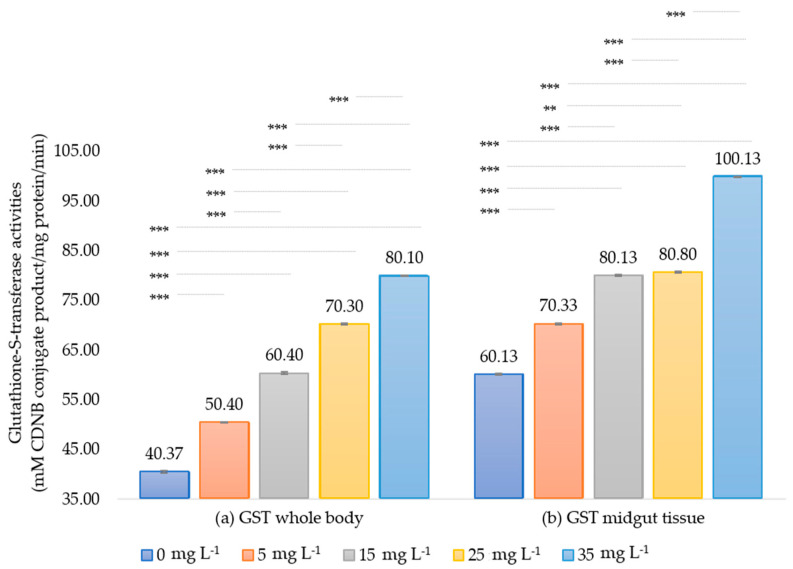
Glutathione-S-transferase enzyme activity (mM CDNB conjugated product/mg protein/min) in the whole larvae body of the first instar (**a**) and midgut tissue of the fifth instar (**b**) of *Bombyx mori* after 24 h of exposure to various concentrations of neem extracts. The bar graph represents means ± SD. Differences between groups were analyzed using one-way ANOVA, followed by Tukey’s HSD post hoc analysis for multiple comparisons. Significant differences between groups are represented as ** and *** for *p* -values < 0.01 and < 0.001, respectively.

The enzymatic activities of all extract treatments were higher than the control. GST activities in the whole body of the first instar larvae were measured as 40.60 ± 0.00, 50.40 ± 0.10, 60.40 ± 0.20, 70.30 ± 0.20, and 80.10 ± 0.10 mM CDNB conjugated product/mg protein/min when exposed to 0, 5, 15, 25, and 35 mg L^−1^ of the extract, respectively. These values correspond to relative activity increases of 1.24, 1.49, 1.73, and 1.97-fold compared to the control, respectively. In the midgut tissue of the fifth instar larvae, the activities were 60.13 ± 0.15, 70.33 ± 0.12, 80.13 ± 0.15, 80.80 ± 0.10, and 100.13 ± 0.06 mM CDNB conjugated product/mg protein/min at the same extract concentrations, respectively. These values indicate relative activity increases of 1.17, 1.33, 1.34, and 1.67-fold compared to the control, respectively (Figure 4). These data demonstrate significant induction of GST activities (*p* < 0.05) in both enzyme sources of silkworm larvae post-treatment. Additionally, significant differences in enzyme activity were observed between the highest (35 mg L^−1^) and lowest (5 mg L^−1^) extract concentrations, with GST activity showing greater induction in the whole body compared to the midgut tissue.

#### 3.2.3. Total Protein Content

**Figure 5:**
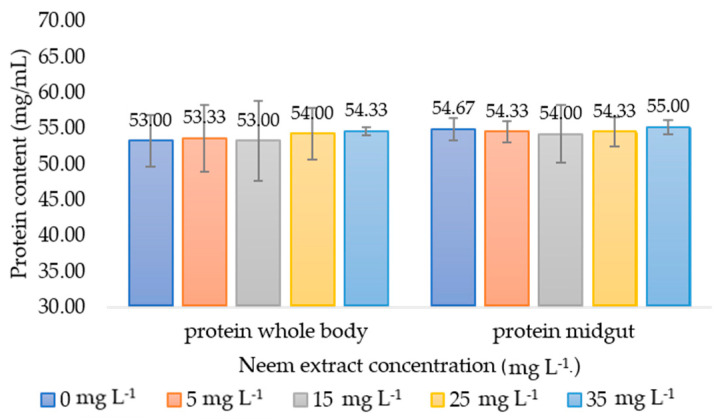
Protein content (mg/mL) in the whole larvae body of the first instar and midgut tissue of the fifth instar of *Bombyx mori* after 24 h of exposure to various concentrations of neem extracts. The bar graph represents means ± SD. Differences between groups were analyzed using one-way ANOVA, followed by Tukey’s HSD post hoc analysis for multiple comparisons.

Using Bradford’s method, protein levels were determined to be 53.00–54.33 mg/mL in the whole body and 54.67–55.00 mg/mL in the midgut tissue of the first instar and fifth instar larvae, respectively, as shown in Figure 5. No significant differences in protein content were observed. This indicates that the enzymatic expressions in both the whole body and midgut tissue of the silkworm larvae occurred without any significant variations in protein levels.

## 4. Discussion

The present study investigated the toxicity of aqueous neem seed crude extract and its effects on the detoxification enzymes, EST and GST, in the larvae of Thai hybrid silkworm strains. The findings revealed that the neem seed crude extract exhibited significant toxicity to silkworm larvae. Mortality rates were assessed across different larval instars, demonstrating a clear dose–response relationship influenced by both extract concentration and exposure duration. The first instar larvae were the most susceptible to the extract, with significant death rates (*p* < 0.05) exceeding 50% at 24 h and reaching 94% at 72 h post-treatment with 15 mg L^−1^. By 72 h, larval mortality peaked at 100% under the 25 mg L^−1^ extract concentration. In stark contrast, the fifth instar larvae showed the lowest susceptibility to the extract as shown in Figure 1, suggesting an increased tolerance to the toxic effects of the extract as the larvae matured. This stage-specific mortality suggests differential susceptibility possibly linked to the developmental stage, cuticle thickness, or detoxification capability of the larvae. Foreign substances commonly have a greater virulence to early instar lepidopteran insects [45,46]. Theoretically, early instar larvae are more vulnerable to xenobiotics due to their thinner epidermis, which allows for easier penetration. Older larvae have thicker waxy layers and higher melanin content, reducing xenobiotic infiltration [47]. Insects also regulate detoxifying enzymes to counteract toxins [30]. The reduced toxicity of neem extract in older larvae might be due to these enzyme systems, as discussed below.

Mortality rates are dose–time-dependent, with higher doses and longer durations causing faster and higher mortality—a theoretical relationship [48]. Our results elucidated that higher doses and prolonged exposure increase toxicity across all larvae stages, with 72 h exposures showing greater toxicity than 24 or 48 h exposures. The toxicity of the extract was further confirmed by the calculated LC_10_, LC_50_, and LC_90_ values. The third instar larvae were found to be highly susceptible to the extract at the LC_10_ level, while the first instar larvae were most affected at the LC_50_ and LC_90_ levels (Figure 2, Table 1). These findings corroborate previous studies that have demonstrated the insecticidal properties of neem-based compounds against various insect pests [18]. The neem extracts significantly increased larval mortality, indicating their toxicity, which may be caused by azadirachtin [49,50]. Higher doses of neem or azadirachtin increased lepidopteran larval mortality and inhibited their development [51]. The maximum azadirachtin (56%) significantly increased silkworm mortality with prolonged exposure (16 days after spraying) [52]. Neem oil and azadirachtin caused 10.80% and 11.60% mortality of silkworms, respectively [53]. Conversely, azadirachtin sprayed on mulberry leaves reported 0% silkworm mortality [54], differing from our findings where mortality never reached zero within 72 h. These findings underline the pronounced toxic effect of neem seed extract on the early larval stages, while later stages such as the fifth instar exhibited reduced vulnerability. This stage-specific response could be attributed to variations inherent to each developmental stage, as mentioned above. In other lepidopteran larvae, azadirachtin feeding caused higher mortality in *Plutella xylostella* L. compared to other neem limonoids from *A. indica* [55]. It also showed toxic feeding rather than antifeedant effects on the larvae of *Helicoverpa armigera* (Hübner) and the cluster caterpillar, *Spodoptera litura* (F) [56]. Neem oil fed to the coffee leaf miner, *Leucoptera coffeella*, resulted in 96.7% mortality [57]. The damage in silkworms exposed to other xenobiotics through the ingestion of contaminated food is demonstrated in the following examples; the λ-cyhalothrin exhibited extreme toxicity to silkworm larvae, resulting in 50 and 86.90% mortality when administered at the LC_50_ value on mulberry leaves and an artificial diet, respectively [30]. Acetamiprid exhibited high toxicity to the fifth instar silkworm larvae, with an LC_50_ (24 h) of 1.5 mg/L [31]. Exposure to sublethal doses of tolfenpyrad in the fifth instar silkworm larvae can influence body weight, development time, cocooning rate, eclosion rate, and pupation rate [11].

In essence in the context of agricultural pest management, the high toxicity of neem extracts against silkworms is a potential concern. Indiscriminate application of neem-based insecticides in or near sericulture areas could pose risks to silk production and economic losses. Conversely, the findings suggest that neem may be an effective biological control agent against silkworm pests, provided adequate safety measures are implemented. Understanding the interplay between xenobiotic metabolism and toxicity could inform the development of sustainable pest control strategies that leverage the insecticidal potential of neem while minimizing the risks to beneficial insects.

In response to the toxic effects of Thai neem seed crude extract, significant changes in detoxification enzyme activities were observed in the whole larvae body and midgut tissue of the first and fifth instar larvae of silkworms, respectively. The enzyme sources from different parts of the body contain various functional and regulatory mechanisms that influence enzyme activity. Previous studies have shown that EST and GST activities are inversely correlated with the tissues of larval instars of silkworms [31,58]. EST activity was significantly reduced in both the whole body and midgut tissue, with greater suppression observed in the whole body (Figure 3), suggesting a disruption in the detoxification mechanism. The suppression of EST activity found in this study was dose-dependent, with higher extract concentrations causing more substantial decreases. Additionally, our experiments indicate that neem extract reduces EST activity and increases silkworm mortality. This suggests the important role of EST in neem extract detoxification and indicates that silkworms are susceptible to this substance.

Theoretically upon xenobiotic entry into the body, insects activate their defense systems by altered metabolization of toxin compounds through detoxification enzymes. Normally, phase I reactions metabolize xenobiotics by enzymatic activity. EST is crucial for defense mechanisms, xenobiotic metabolism, and the development of resistance to both synthetic and natural toxins [59]. The activity levels of EST are positively correlated with insecticide exposure, highlighting its role in insecticide detoxification [60]. Although no reports have been found yet on neem extract affecting EST enzymes in silkworms, however, previous studies have stated that limonoids, the secondary metabolites from Meliaceae plant family and the same active ingredients in neem, are effective against lepidopteran insects [28]. For example, the extracts from senescent leaves of *M. azedarach* inhibit EST activity in *S. frugiperda* in vitro [61]. Likewise, the *M. toosendan* extract showed inhibited midgut EST activity in *S. litura* [62]. Our results aligned with the previous other reported that the midgut tissue EST activity was less profoundly affected by neem extract compared to whole-body silkworm larvae. This suggests that EST is more involved in neem extract metabolism in the midgut than in the whole body of silkworm larvae.

In principle, the insect midgut, including silkworm midgut, is the main digestive system that plant leaves first enter. It is not only for nutrient breakdown but also for destroying xenobiotics that contaminate food. These are metabolized by detoxifying enzymes, activated to be neutralized and expelled, maintaining overall physiological balance [63]. EST is predominantly synthesized in the midgut [11,23]. After treatment with neem extract, the observed decrease in EST activity suggests a reduction in toxin metabolism in the insect. This is likely due to the toxic effects of allelochemical derivatives on active sites, modulation of gene expression, or oxidative stress, which impairs their normal function [64]. The reduction in EST levels at higher concentrations of neem extract indicates a decrease in enzyme levels due to extract-induced stress [18]. Changes in the physio-biochemical balance of the midgut may also impact enzyme activity. Thus, the reduction in EST activity following neem extract treatment suggests that these substances affect gut physiological processes, influencing enzyme activity. The reduced EST activity impairs detoxification by decreasing the ability to break down harmful substances, leading to the accumulation of toxins and cell damage in the midgut [65]. This leads to decreased digestive efficiency, causing insects to have difficulty digesting and absorbing nutrients, which can result in malnutrition [66]. Additionally, changes in the enzyme activity and gut pH disrupt the microbial community, causing an imbalance in the gut microbiota, which further impairs digestion and detoxification processes [67].

We determined that the present study provides evidence for the inhibitory effects of neem seed extract on esterase enzyme activity in silkworms. The suppression of EST activity in both whole-body larvae and midgut tissue, with a more pronounced effect in the whole body, highlights the potential of neem-based compounds as natural insecticides. Further research is needed to elucidate the specific mechanisms underlying the inhibition of EST activity and to explore the implications of these findings for the development of effective and sustainable pest management strategies.

GST, a Phase II detoxifying enzyme, is important for the detoxification system. Our results found that GST activity is inversely related to EST activity. When both instar silkworm larvae consume mulberry leaves contaminated with neem extract, the GST exhibits an increase in both the midgut and the whole body, with a more pronounced increase in the whole body. In silkworms, typical chemical insecticides induce GST activity in the fat body [36,58,68] and midgut [31,40,58] were reported. Phytochemicals like quercetin also increase GST activity in both the whole body and midgut tissue at low concentrations [69].

Our study found that GST activity is significantly upregulated in both larval stages at higher neem extract concentrations, reaching a 1.97-fold increase at 35 mg L^−1^ (Figure 4). This suggests a detoxification role for GST in both the whole body and midgut tissue. The increased GST activity is a defensive response to the potential toxicity of the extract and may also impact the survival of the silkworms, as indicated by our bioassay results. This upregulation of GST activity is a common defense mechanism employed by insects to mitigate the toxic effects of xenobiotics, including plant-derived compounds [70,71]. The observed induction of GST activity in the silkworm larvae suggests a potential detoxification response to the neem seed extract. In principle, enhanced GST activity contributes to insecticide tolerance in insects [72]. Notably, neem extracts more strongly activated GST in the whole body, with activity increasing proportionally to the extract concentration. In general, GST enzymes play important roles in detoxification processes in many organisms, including insects and other arthropods during their larval stages. Various chemical exposures can induce their activity. Quercetin (1%) showed the highest induction activities of GST in the whole body of silkworm larvae, observed to be 2.6-fold towards CDNB [69]. The silkworm larvae fed with phoxim at a concentration of 4.0 mg/mL for 24 h, GST activity did not change in the midgut and, conversely, significantly increased in the fat body [40].

Several mechanisms may mediate the increased GST activity in silkworm larvae exposed to neem extract. Firstly, neem allelochemicals might activate transcription factors, such as Nrf2, which bind to the promoter regions of GST genes and increase their expression. This transcriptional upregulation leads to an increase in the production of GST enzymes, enhancing the detoxification capacity of larvae [71]. Secondly, neem extract may induce the expression of specific GST isoforms that are more efficient in detoxifying the allelochemicals present in neem. This selective induction of specific GST isoforms allows the larvae to better cope with the specific toxins encountered in their diet [73,74]. Furthermore, the midgut is the primary site of contact and absorption for neem allelochemicals, which may explain the lesser increase in GST activity observed in this tissue compared to the whole body [28,71]. The increased GST activity in response to neem extract is an adaptive mechanism that helps silkworms cope with the potential toxicity of the phytochemicals. The effectiveness of this detoxification response in promoting silkworm survival depends on the concentration of neem extract and the duration of exposure. At low to moderate concentrations, the increased GST activity may be sufficient to detoxify the neem allelochemicals, allowing the silkworms to survive. However, the allocation of resources towards detoxification may affect growth and silk production, as the larvae must balance their energy expenditure between detoxification and other vital processes [36].

We deduced that neem extract increases GST activity in both the midgut and whole body of the first and fifth instar silkworm larvae, with a more pronounced increase in the whole body. The increase in GST activity is a defensive response aimed at detoxifying the neem allelochemicals and promoting larvae survival. The ultimate impact on silkworm survival depends on the neem extract concentration and efficiency of the detoxification responses.

Lastly, the relationship between protein content and detoxification enzyme activity is significant. Our study found no difference in the total protein content of both enzyme sources (Figure 5). EST activity decreased while GST activity increased after exposure to the extract (Figure 3 and Figure 4), indicating complex enzyme regulation [75,76]. Increased GST activity with normal protein levels shows enhanced detoxification independent of protein synthesis [77,78]. This underscores the complexity of biochemical responses and the importance of protein integrity and stimulating factors for effective detoxification [79]. Therefore, enzyme activity studies should measure total protein to understand its influence on enzyme expression.

The present study provides valuable insights into the toxic effects of Thai neem seed crude extract on silkworm larvae and the associated modulation of detoxification enzyme activities. The findings contribute to our understanding of the biochemical responses of Thai hybrid silkworm strains to neem-based insecticides. However, the limitation of our study lies in assessing only the activity of two enzymes from two tissue sources in two larval stages. The need to understand the broader implications of whether these enzymes can effectively detoxify neem extract remains. Therefore, further research is needed, considering that other organs related to toxin accumulation and release, such as fat body tissue and Malpighian tubules, as well as other detoxifying enzymes, are also crucial for detoxification and are being further investigated in our study. For instance, cytochrome P450 plays a crucial role in degrading toxic substances and is essential for the elimination of insecticides. Although not studied in this research, it has been reported that these enzymes are vital for insect detoxification, especially in processing plant secondary metabolites [80]. They become activated as larvae develop, potentially leading to lower sensitivity to treatments with neem and other secondary metabolites from Meliaceae plants in larger Lepidopteran larvae [28]. Cytochrome P450 acts as a catalyst in the oxidative transformation of compounds, which is considered important among phase I reactions. During this process, substrates are hydroxylated, leading to decreased biological activity and degradation of toxins [81,82].

## 5. Conclusions

These results highlight the extract’s impact on larval mortality and enzyme activity. These novel findings suggest that Thai neem seed crude extract exhibits toxicity to these silkworm strains, although the late larval stage plays a key role in detoxification. Therefore, careful consideration is required, especially with young larvae, to ensure safety in sericulture.

## Figures and Tables

**Table 1:** Lethal concentrations in terms of LC_10_, LC_50_ and LC_90_ of neem extracts (mg L^−1^) against *Bombyx mori* larvae at 24, 48, and 72 h after treatment.

Larval Stage (Instar)	Time Exposure (h)	Slope ± SE	Intercept ± SE	Toxicity Value of Neem Extract (mg L^−1^)		
				LC_10_ (CI)	LC_50_ (CI)	LC_90_ (CI)
1	24	2.68 ± 0.34	(−2.7252) ± 0.40	3.46 (1.50–14.00)	10.00 (3.61–63.00)	31.00 (8.64–281.00)
	48	2.76 ± 0.34	(−2.2296) ± 0.36	2.21 (1.13–6.70)	6.43 (2.67–28.00)	19.00 (6.27–118.00)
	72	3.74 ± 0.38	(−2.6904) ± 0.34	2.38 (1.43–5.22)	5.23 (2.74–14.00)	12.00 (5.24–39.00)
2	24	2.19 ± 0.24	(−2.309 ± 0.30	2.95 (1.42–9.50)	11.00 (4.26–55.00)	44.00 (13.00–318.00)
	48	2.32 ± 0.25	(−2.0278) ± 0.29	2.10 (1.11–5.69)	7.47 (3.14–29.00)	27.00 (8.87–152.00)
	72	3.07 ± 0.31	(−2.223) ± 0.30	2.02 (1.21–4.41)	5.29 (2.69–15.00)	14.00 (5.96–50.00)
3	24	1.53 ± 0.16	(−1.7105) ± 0.20	1.91 (1.01–5.07)	13.00 (4.95–60.00)	90.00 (24.00–700.00)
	48	1.57 ± 0.15	(−1.4100) ± 0.18	1.21 (0.73–2.54)	7.88 (3.51–26.00)	51.00 (17.00–277.00)
	72	2.03 ± 0.26	(−1.5737) ± 0.30	1.39 (0.74–4.06)	5.96 (2.36–29.00)	25.00 (7.45–211.00)
4	24	1.81 ± 0.13	(−2.0759) ± 0.16	2.74 (1.72–5.03)	14.00 (7.23–33.00)	71.00 (30.00–216.00)
	48	1.91 ± 0.14	(−1.8987) ± 0.17	2.10 (1.32–3.93)	9.83 (5.06–24.00)	46.00 (19.00–148.00)
	72	2.00 ± 0.15	(−1.7668) ± 1.77	1.75 (1.12–3.18)	7.61 (4.03–18.00)	33.00 (14.00–104.00)
5	24	1.85 ± 0.13	(−2.4166) ± 0.17	4.10 (2.40–8.32)	20.00 (9.71–53.00)	99.00 (39.00–338.00)
	48	1.89 ± 0.13	(−2.1709) ± 0.16	2.96 (1.81–5.70)	14.00 (7.13–35.00)	68.00 (28.00–218.00)
	72	1.92 ± 0.14	(−1.9420) ± 0.17	2.21 (1.39–4.12)	10.00 (5.30–25.00)	48.00 (20.00–152.00)

Mortality rates of different instars of *Bombyx mori* larvae exposed to various concentrations of neem extracts were analyzed using probit analysis at 24, 48 and 72 h. Lethal concentrations (LC) at 10, 50 and 90% were presented with 95% confidence intervals.

## Data Availability

Data are available on request from the corresponding author, A.R., upon reasonable request.
